# Effect of the age and sex on growth performance and feather quality of 13 to 25-weeks-old White Roman geese

**DOI:** 10.1016/j.psj.2023.102941

**Published:** 2023-07-19

**Authors:** M.J. Lin, S.C. Chang, L.J. Lin, S.Y. Peng, T.T. Lee

**Affiliations:** ⁎ Changhua Animal Propagation Station, Livestock Research Institute, Council of Agriculture, Executive Yuan, Changhua 52149, Taiwan; † Kaohsiung Animal Propagation Station, Livestock Research Institute, Council of Agriculture, Executive Yuan, Pingtung 91201, Taiwan; ‡ School of Chinese Medicine, College of Chinese Medicine, China Medical University, Taichung 40402, Taiwan; § Department of Animal Science, National Pingtung University of Science and Technology, Pingtung 91201, Taiwan; ‖ Department of Animal Science, National Chung Hsing University, Taichung 40227, Taiwan; ¶ The iEGG and Animal Biotechnology Center, National Chung Hsing University, Taichung 40227, Taiwan; # Smart Sustainable New Agriculture Research Center (SMARTer), Taichung 40227, Taiwan

**Keywords:** goose, feathers quantity, down, gender, age

## Abstract

This study aimed to determine the effect of age and sex in weeks on growth, carcass performances, and feather quality of White Roman geese and analyze the relative effect of age and sex in weeks on feather characteristics in geese. In animal experiments, 120 White Roman geese aged 13 wk were randomly distributed among 12 pens and fed grower diet ad libitum during the growing period, with each pen containing 10 males or 10 females, depending on a completely random design. Ten geese each in their 13, 15, 17, 19, 21, 23, and 25 wk of age were sacrificed. The results showed that the age had no significant effect on the body weight (**BW**) of geese (*P* > 0.05). The chest girth was significantly longer in 21 to 25 wk old than in 13- to 19-wk-old geese (*P* < 0.05). The results reveal no significant change in carcass weight of grower geese among 13- to 25-wk age groups (*P* > 0.05). The down percentage of the 25-wk-old age group was significantly higher than that of the 13-, 15-, and 17-wk age groups (*P* < 0.05). The male geese had a higher dry feather weight than the females (*P* < 0.05). Similarly, the 18-h feed-deprived body weight (**18-h FDBW**) and 4 to 10 cm feather weight were significantly negatively correlated in grower geese (−0.42). This provides the feather industry an opportunity for the better utilization of grower geese by-products. In conclusion, the age has no influence on BW among 13- to 25-wk-old geese, and a good down percentage and dry weight were observed at 25 wk of age. The 18-h FDBW and down percentage had a significantly negative correlation (−0.55) in grower geese.

## INTRODUCTION

The domestic goose shows an obvious seasonal pattern of reproductive activity under natural lighting conditions (Zeman et al., 1990). Their reproductive seasons start in late autumn in subtropical areas or early spring in temperate areas (Gillette, 1976; Chang et al., 2016, 2023). In Landaise gray goose, laying intensity rises from 0 in January to 50% in April to May (Mialon et al., 2004).

The yields of down and feather, which are natural substances for keeping warm in winter, are of economic importance in the goose industry (Chang et al., 2023). Goose feathers and down provide high-quality insulation in garments and fabrics. Goose down has a cross-sectional hollow geometry and is more compression resistant than duck down (Fuller, 2015).

Nagy et al. (2011) indicated that the heritability of the feather production ability is relatively low (*h*2 = 0.35). The yield of feathers and down from each manual harvest amounts to 80 to 120 g per goose, depending upon the frequency and degree of completeness of the harvesting. Farrell (2004) indicated that a 5 kg goose would produce up to 400 g of feather and down per year. Although feathers are known for their lightness, a bird's plumage weighs circa 2 to 3 times more than its skeleton and about 5 to 7% of the total body weight (Del Hoyo et al., 1992). In one study, hen and turkey feather weights were reported to be 80 to 100 g or 190 to 300 g, individually, and which did not yield down in land poultry. Another study reported a feather yield of 250 to 300 g per goose and the down weight of about 50 to 60 g and 20% of feather weight (Szado et al., 1995).

Saatci (2008) showed that geese at 10 wk had heavier feathers than those at 12 wk, while geese at 12 wk produced more down. Van De Wetering and Cooke (2000) indicated that the mean daily change in primary feather length was 2.6%, which is consistent with rates reported for other waterfowl species. After slaughter, 90 to 220 g of marketable feathers can be obtained per goose from 9 to 30 wk of age (Schneider, 1995; Kozák, 2011). Kozák et al. (1997) indicated that the correlation between the number of raw feathers and the percentage of down is significant, but with a negative regression value in the case of males. According to Yildiz et al. (2009), there are intensive vexilla in females because of their barbs which are thinner than those the males.

In Europe, the harvesting of feathers from fattening geese kept on pastures and from breeder duck and geese flocks in the nonlaying period can be an additional source of income (Pingel, 2010). Toth et al. (2012) indicated that the handling of geese causes an elevation in plasma corticosterone level, while feather gathering does not result in a higher corticosterone level than the handling or catching of the bird. Kozák et al. (2010) indicated that during the course of slaughter and processing, feather and down are exposed to various unfavorable effects that degrade their quality. Conversely, hand-harvested feather and down harvested from live geese are superior to the industrial feather product in several respects. In Taiwan, animal welfare is highly valued; techniques such as live feather plucking are prohibited, and goose feathers or down are generally harvested after slaughter and then subsequently cleaned and processed. Therefore, to raise the quality and quantity of goose feathers, the age of geese and their sex needs to be monitored. To our knowledge, little information is currently available regarding the influence of age and sex on feather composition. Therefore, the objective of this study was to investigate the influence of age and sex in weeks on growth, feather weight, and down characteristics of grower geese to improve feather quality while making effective use of the feeding scheme.

## MATERIALS AND METHODS

### Animals and Experimental Design

One hundred twenty White Roman geese aged 13 wk were distributed among 12 pens randomly, with each pen containing 10 males or 10 females depending on a completely random design (**CRD**), and fed grower diet ad libitum during the growing period. Ten geese each in their 13, 15, 17, 19, 21, 23, and 25 wk of age were sacrificed (each week, 1 male or 1 female of each pen was sacrificed, with a total of 12 geese at 2-wk intervals). The care and use of all geese were according to the Regulations of Laboratory Animals and approved by the Institutional Animal Use and Care Committee (**IAUCC** 10208) according to the Regulations of Laboratory Animals, Changhua Animal Propagation Station, Livestock Research Institute (located at 23°51′N and 120°33′E), COA-LRI, Council of Agriculture, Taiwan. During the experimental period, the geese experienced a natural photoperiod and day length of 11.0 to 12.0 h. The dimension of the finishing house pen was 3.95 × 2.5 m (9.88 m^2^), and the rearing density was 1.01 m^2^ per goose. It had a cement floor, one tank, and water dispensers; the drinking water was provided ad libitum. During the experimental period, the geese were fed a grower diet (Table 1) formulated to meet the nutrient requirements according to the National Research Council (NRC, 1994).

**Table 1:** Ingredients and nutrient compositions of the experimental diets (as-fed basis).

Ingredients	%
Corn	64.3
Soybean meal, 44%	21.5
Wheat bran	5.0
Cane molasses	3.0
Salt	0.3
Dicalcium phosphate	0.8
Limestone, pulverized	1.60
Choline chloride, 50%	0.1
DL-methionine	0.2
Rice bran	3.0
Vitamin premix^1^	0.1
Mineral premix^2^	0.15
Total	100.0
Calculated values	
ME, kcal/kg	2800
Crude protein, %	15.5
Calcium, %	0.73
Phosphorus, %	0.64

1 Vitamin premix: Each kg contained vitamins A 10,000,000 IU, D3 2,000,000 IU, E 20,000 IU, B1 1 g, B2 4.8 g, B6 3 g, B12 0.01 g, biotin 0.2 g, K3 1.5 g, D-calcium pantothenate 10 g, folic acid 0.5 g, nicotinic acid 25 g.

2 Mineral premix: Each kg contained Cu 15.0 g, Fe 80 g, Zn 50 g, Mn 80 g, Co 0.25 g, I 0.85 g.

### Growth Performance and Carcass Characteristic

At 13, 15, 17, 19, 21, 23, and 25 wk of age, the performance of the geese was assessed by measuring the body weight (**BW**), and the BW gain and feed conversion ratio were recorded. On the same day, 12 geese (6 males and females each) per pen were randomly selected and then sacrificed by exsanguination. The samples from the carcass were weighed and harvested individually and then expressed in grams per goose.

### Sample Preparation

After the geese are slaughtered, the feathers obtained after depilation are called total feathers. The total feather was washed with water for 1 h and dried in a dryer at 120°C for 6 min. The dry treatment can be added to the cleaned down and feathers. After that, the hydrophobic treatment can render them to be more hydrophobic. The dried feathers were placed in an oven at 105°C for drying. Afterward, this treatment obtains feather air-dried weight. The feathers were weighed and manually categorized into 10 cm or more, 4 to 10 cm, and less than 4 cm, and then weighed, respectively.

The feathers were cleaned, and those less than 4 cm in length were weighed by analyzing down feathers to obtain the down weight of each goose. The samples were thoroughly mixed and sampled. The delicate feather was taken after the impurities were processed by the sanding machine. The test on delicate feathers was carried out by the quartering method, taking 10 to 15 g for assay. The delicate feather is divided into goose down, small feather (less than 6.5 cm), waste silk, shell silk, and residual matter, and its weight were recorded.

### Statistical Analyses

The data of the variables collected were statistically analyzed using the general linear model procedure of SAS software following a random arrangement.

The mathematic model was:

$$Y_{ijk}=T_{i}+S_{j}+T\times S_{k}+\varepsilon_{ijk}$$

where, $Y_{ijk}$ is the observed response of bird in a pen; *µ* is the overall mean; $T_{i}$ is the fixed effect of the age; $S_{j}$ is the fixed effect of sex; $T\times S_{k}$ is the fixed effect of sex and age interaction; $\varepsilon_{ijk}$ is the residual error when the pen was regarded as an experimental unit, $\varepsilon_{ijk}∼N\left(0,\sigma_{\varepsilon }^{2}\right)$.

The mean values for the results of 7 wk of age and sex were compared using the LSMEANS with a significant level of *P* < 0.05. According to the analysis of variance (**ANOVA**), we further proceeded to develop the regression of the variable of interest on the week level. Multivariate ANOVA was carried out also to understand the partial correlations between the variables measured.

## RESULTS

### Growth Performance

The effects of age and sex in weeks on growth performances in grower geese are mentioned in Table 2. The age had no significant effect on BW (*P* > 0.05). The back length of geese was significantly longer at 17- to 25-wk-old than of the 13- and 15-wk-old group (*P* < 0.05). The chest girth was significantly longer in the 21- to 25-wk-old group than in the 13- to 19-wk-old group (*P* < 0.05). The BW, back length, sternum length, and chest girth of the male geese were significantly higher than that of the females during 13- to 25-wk-old (*P* < 0.05).

**Table 2:** The effect of age and sex on growth performances of grower geese.^1^

	Age in weeks								Sex in weeks			*P* value		
Item	13	15	17	19	21	23	25	SEM^2^	M	F	SEM	A	S	A × S
Body weight, kg/bird	5.21	5.46	5.71	5.77	5.66	5.54	5.42	0.1751	5.83^x^	5.25^y^	0.0919	0.3155	0.0001	0.3624
Back length, cm/bird	29.9^c^	30.6^bc^	31.4^ab^	31.4^ab^	31.9^a^	31.8^a^	31.8^a^	0.2879	31.8^y^	30.8^y^	0.1511	0.0002	<0.0001	0.3512
Sternum length, cm/bird	17.2^e^	18.8^bc^	18.1^d^	18.9^ab^	18.8^bc^	19.4^a^	18.9^ab^	0.2025	19.0^y^	18.1^y^	0.1063	<0.0001	<0.0001	0.4001
Chest girth, cm/bird	47.0^d^	50.4^c^	51.8^bc^	52.7^b^	55.0^a^	56.5^a^	56.4^a^	0.7681	53.9^y^	51.8^y^	0.4032	<0.0001	0.001	0.4107

a–e Different superscript letters in rows mean a significant difference between averages for age (*P* < 0.05).

x,y Different superscript letters in rows mean a significant difference between averages for sex (*P* < 0.05). M: male. F: female. A: age in weeks. S: sex in weeks. A × S: age × sex interaction.

1 Results are given as the means of 6 pens for 10 geese each pen.

2 SEM = standard error mean.

### Carcass Performances

The effects of age and sex in weeks on carcass performances in grower geese from wk 13 to 25 are mentioned in Table 3. The carcass weight of grower geese exhibited no significant difference in the 13- to 25-wk age groups (*P* > 0.05). The legs weight percentage of 18-h feed-deprived body weight (**FDBW**) of the 13-wk age group was significantly higher than that of the 15- to 21-wk age groups (*P* < 0.05). The paw weight, heart weight, and the percentage of paw weight and heart weight of FDBW in the male group were significantly higher than that in the female group in the 13- to 25-wk age groups (*P* < 0.05). In the 13- to 25-wk age group, the intestinal weight percentage of FDBW of the male group was significantly lower than that of the female group (*P* < 0.05).

**Table 3:** The effect of age and sex on carcass performances of grower geese.^1^

Item	Age in weeks								Sex in weeks			*P* value		
	13	15	17	19	21	23	25	SEM^2^	M	F	SEM	A	S	A × S
Eighteen-h feed-deprived body weight, kg/bird	4.84	5.18	5.43	5.29	4.88	5.20	5.20	0.168	5.23	5.06	0.132	0.3619	0.3618	0.2543
Carcass weight, kg/bird	3.85	4.04	4.29	4.15	3.90	4.21	4.07	0.132	4.18	3.96	0.103	0.4023	0.1561	0.2393
Head and neck weight, g/bird	497	459	497	505	481	470	491	18.3	507	464	14.4	0.7397	0.0554	0.3864
Back weight, g/bird	815	949	1026	981	894	1027	941	42.5	966	929	33.2	0.0854	0.4417	0.1788
Breast weight, g/bird	645	708	788	784	736	827	784	36.5	775	731	28.6	0.1428	0.2939	0.9370
Breast muscle weight, g/bird	289	266	278	234	239	245	185	28.7	254	243	22.5	0.6546	0.7291	0.9279
Legs weight, g/bird	693	704	716	693	643	735	740	26.89	713	693	21.0	0.7692	0.1410	0.3569
Wing weight, g/bird	588	620	602	558	575	558	586	21.6	612	556	16.9	0.6914	0.0372	0.8427
Paw weight, g/bird	153	157	147	144	147	140	156	6.99	161^x^	137^y^	5.48	0.8585	0.0089	0.1262
Abdominal fat pad weight, g/bird	164	180	237	252	187	208	183	21.5	194	209	16.8	0.2670	0.5462	0.5720
Liver weight, g/bird	78.7	75.4	81.8	89.75	67.5	79.5	69.5	5.81	79.6	75.3	4.55	0.6979	0.5085	0.3404
Heart weight, g/bird	34.3	36.8	39.8	36.5	37.0	40.3	36.0	1.04	40.9^x^	33.5^y^	0.811	0.052	<0.0001	0.0024
Gizzard weight, g/bird	196	186	205	173	188	184	184	9.42	186	189	7.37	0.647	0.7972	0.4833
Intestinal weight, g/bird	244	291	318	318	230	248	280	19.6	257	295	15.4	0.1567	0.1050	0.5236

x,y Different superscript letters in rows mean a significant difference between averages for sex (*P* < 0.05). M: male. F: female. A: age in weeks. S: sex in weeks. A × S: age × sex interaction.

1 Results are given as the means of 6 pens for 10 geese each pen.

2 SEM = standard error mean.

### Feather Performances

The effects of age and sex in weeks on feather performances in grower geese at 13 to 25 wk are listed in Table 4. There was no significant difference among the 13- to 25-wk age groups on >10 cm feather weight of grower geese (*P* > 0.05). The weight of 4 to 10 cm feathers in the 25-wk age group was significantly heavier than that of the 13- to 21-wk age groups (*P* < 0.05). The dry feather weight percentage of >10 cm feathers of the 13-wk age group was significantly higher than that of the 15- to 25-wk age groups (*P* < 0.05). The dry feather weight percentage of 4 to 10 cm feathers of the 25-wk age group was significantly higher than that of the 13- to 19-wk age groups (*P* < 0.05). The dry feather weight percentage of <4 cm feathers of the 13-wk age group was significantly higher than that of the 17-, 21-, 23-, and 25-wk age groups (*P* < 0.05). The down percentage of the 25-wk age group was significantly higher than that of the 13-, 15-, and 17-wk age groups (*P* < 0.05).

**Table 4:** The effect of age and sex on feather quality of grower geese.^1^

	Age in weeks								Sex in weeks			*P* value		
Item	13	15	17	19	21	23	25	SEM^2^	M	F	SEM	A	S	A × S
Live body weight, kg/bird	5.19	5.49	5.79	5.29	4.88	5.41	5.20	0.311	5.51	5.28	0.140	0.1683	0.7469	0.6846
>10 cm feather weight, g/bird	72.7	70.5	78.8	77.4	73.0	78.0	79.7	2.824	82.5^x^	69.0^y^	2.209	0.4080	0.0002	0.3212
4–10 cm feather weight, g/bird	60.5^d^	85.0^c^	100^bc^	103^b^	101^bc^	115^ab^	139^a^	4.285	108^x^	92.8^y^	3.353	<0.0001	0.0034	0.0902
<4 cm feather weight, g/bird	87.5	89.7	94.6	98.9	86.1	102	105	3.243	97.1	92.7	2.538	0.0751	0.2486	0.3522
Wet feather weight, g/bird	431^c^	480^b^	519^b^	523^b^	475^bc^	548^b^	637^a^	28.06	536^x^	497^y^	12.68	<0.0001	0.0339	0.1299
>10 cm feather weight, % of Dry feather weight	33.2^a^	28.8^b^	28.7^b^	27.5^b^	27.9^b^	26.2^b^	24.7^b^	1.534	29.1	27.2	0.693	0.0004	0.0876	0.4918
4–10 cm feather weight, % of Dry feather weight	27.2^d^	34.4^c^	36.6^bc^	37.1^bc^	38.3abc	38.9^ab^	42.7^a^	1.743	36.9	36.0	0.788	<0.0001	0.4652	0.2632
<4 cm feather weight, % of Dry feather weight	39.5^a^	36.8^ab^	34.7^b^	35.4^ab^	33.8^b^	34.9^b^	32.6^b^	1.809	34.1^y^	36.7^x^	0.817	0.0183	0.0342	0.6432
>10 cm feather weight, % of FDBW^3^	0.65^a^	0.53^b^	0.50^b^	0.53^b^	0.57^ab^	0.49^b^	0.47^b^	0.044	0.55	0.52	0.020	0.0035	0.3762	0.6473
4–10 cm feather weight, % of FDBW	0.53^c^	0.64^b^	0.65^b^	0.71^ab^	0.79^a^	0.72^ab^	0.83^a^	0.052	0.70	0.69	0.024	0.0002	0.6486	0.3097
<4 cm feather weight, % of FDBW	0.77^a^	0.68^b^	0.61^b^	0.68^ab^	0.70^ab^	0.64^b^	0.62^b^	0.049	0.64	0.70	0.022	0.0235	0.0938	0.8492
Goose down, %	56.0^d^	60.7^c^	66.4^b^	67.6^ab^	69.9^ab^	72.5^ab^	75.0^a^	2.727	66.1	67.6	1.232	<0.0001	0.4192	0.9604
Small feather, %	40.4^a^	36.1^a^	31.4^b^	23.0^bc^	28.7^bc^	25.2^bc^	22.8^c^	2.813	31.2	30.1	1.271	<0.0001	0.5317	0.9648
Waste silk, %	0.37	0.36	0.43	0.32	0.44	0.46	0.03	0.123	0.38	0.31	0.055	0.3372	0.3873	0.7026
Shell silk R, %	0.27	0.18	0.22	0.27	0.19	0.14	0.02	0.140	0.15	0.22	0.063	0.8781	0.4518	0.5739
Residual matter, %	2.89	2.69	1.52	1.82	0.84	1.85	5.21	0.911	2.98	1.82	0.412	0.0535	0.0663	0.6827

a–d Different superscript letters in rows mean a significant difference between averages for age (*P* < 0.05).

x,y Different superscript letters in rows mean a significant difference between averages for sex (*P* < 0.05). M: male. F: female. A: age in weeks. S: sex in weeks. A × S: age × sex interaction.

1 Results are given as the means of 6 pens for 10 geese each pen.

2 SEM = standard error mean. Waste silk, and residual matter.

3 FDBW: feed-deprived body weight.

The small feather percentage of the 13- and 15-wk age group was significantly higher than that of the other age groups (*P* < 0.05). The dry feather weight, >10 cm feather weight, 4 to 10 cm feather weight, and dry feather weight percentage of FDBW of males were significantly higher than that of the females (*P* < 0.05). The <4 cm percentage of dry feather weight of males was significantly lower than that of the females (*P* < 0.05).

**Figure 1:**
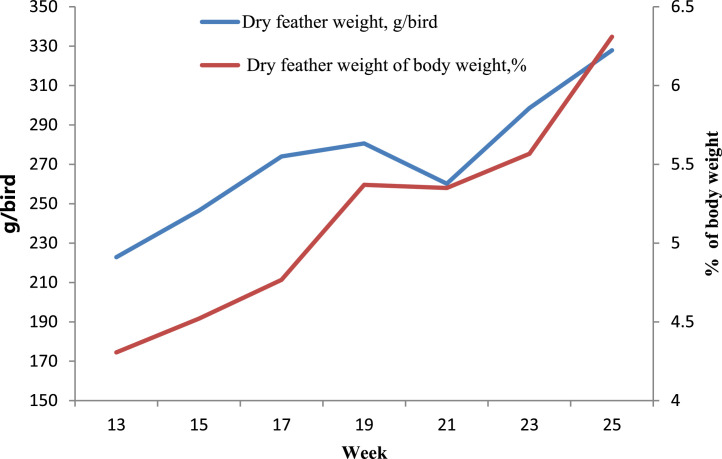
Effect of age on feather weight and percentage of body weight of grower geese.

The effect of age and sex in weeks on feather weight and percentage of BW of grower geese are shown in Figure 1. The dry feather weight and that of dislodged furfur of the 25-wk age group were significantly heavier than that of the 13- to 21-wk age groups (*P* < 0.05). The dry feather weight percentage of FDBW of the 25-wk age group was significantly higher than that of the 13- to 21-wk age groups (*P* < 0.05).

In the males, >10 cm feather weight and 4 to 10 cm feather weight were significantly higher than that of females (*P* < 0.05) (Table 4), while <4 cm feather weight percentage of dry feather weight of males was significantly lower than that of females (*P* < 0.05).

### Correlation Analysis

The effect of correlation coefficients among growth, carcass, and feather characteristics of grower geese are shown in Table 5. The 18-h FDBW and 4 to 10 cm feather weight, dry feather weight percentage of FDBW, 4 to 10 cm feather weight percentage of dry feather weight, exhibited a significantly negative correlation with <4 cm, 4 to 10 cm, and >10 cm feather weight percentage of FDBW and down percentage in grower geese.

**Table 5:** Partial correlation coefficients among growth, carcass, and feather characteristics of grower geese.

Item	BL	SL	BG	FDBW	DFW	L10CFW	L410CFW	L4CFW	PDFW	PL10CM	PL410CM	PL04CM	P10CFW	P410CFW	P04CFW	DOWP
BW^1^	0.58**^2^	0.74***	0.85***	−0.15	−0.18	−0.28	0.02	−0.26	0.03	−0.11	0.15	−0.08	−0.12	0.10	−0.04	−0.15
BL		0.47*	0.57**	−0.19	−0.22	−0.25	0.00	−0.38	0.03	−0.05	0.19	−0.22	−0.04	0.10	−0.09	0.03
SL			0.67***	−0.15	−0.04	−0.22	0.04	0.00	0.09	−0.17	0.08	0.10	−0.07	0.10	0.13	−0.21
CG				−0.16	−0.12	−0.33	0.14	−0.30	0.06	−0.19	0.29	−0.19	−0.17	0.19	−0.07	−0.10
FDBW					−0.11	0.43*	−0.42*	0.12	−0.83***	0.47*	−0.58**	0.25	−0.60***	−0.77***	−0.76***	−0.55**
DFW						0.34	0.85***	0.67***	0.63***	−0.43*	0.57**	−0.29	0.38	0.62***	0.53**	0.26
L10CFW							−0.07	0.07	−0.16	0.69***	−0.36	−0.36	0.46*	−0.27	−0.31	−0.05
L410CFW								0.38	0.80***	−0.69***	0.91***	−0.46*	0.32	0.89***	0.60***	0.36
L4CFW									0.25	−0.49**	0.09	0.51**	−0.07	0.17	0.54**	0.01
PDFW										−0.60***	0.77***	−0.37	0.67***	0.96***	0.87***	0.59**
PL10CM											−0.75***	−0.19	0.17	−0.70***	−0.71***	−0.21
PL410CM												−0.51	0.22	0.91***	0.54**	0.37
PL04CM													−0.55**	−0.45*	0.12	−0.28
P10CFW														0.50**	0.46*	0.52
P410CFW															0.77***	0.54**
P04CFW																0.48*

1 BW: body weight, kg/bird. BL: back length. SL: sternum length, cm/bird. CG: chest girth, cm/bird. FDBW: eighteen-h feed-deprived body weight, kg/bird. DFW: dry feather weight. L10CFW: >10 cm feather weight, g/bird. L410CFW: 4 to 10 cm feather weight, g/bird. L4CFW: <4 cm feather weight, g/bird. PDFW: dry feather weight, % of body weight. PL10CM: >10 cm feather weight, % of dry feather weight. PL410CM: 4 to 10 cm feather weight, % of dry feather weight. PL04CM: <4 cm feather weight, % of dry feather weight. P10CFW: >10 cm feather weight, % of FDBW. P410CFW: 4 to 10 cm feather weight, % of FDBW. P04CFW: <4 cm feather weight, % of FDBW. DOWP: down, %.

2 *: *P* < 0.05. **: *P* < 0.01. ***: *P* < 0.001.

## DISCUSSION

Ochrem et al. (2018) indicated that the first and the subsequent 2 gatherings on down composition were lighter than the third gathering group (79 and 85.1 g vs. 100.7 g). The age of the birds had no effect on the quantity of down. The high stocking density could reduce the growth and impaired feather quality and intestinal development of geese (Liu et al., 2021). Hamadani et al. (2014) indicated an increasing trend of BW with the increase in age. Tilki et al. (2005) showed that the average live weight of male and female Native Turkish geese at 16 wk of age was 4,371 g and 4,071 g, respectively; live weight increased rapidly until 10 to 12 wk of age, and the increase was only a little after 12 wk of age. Until 12 wk, the age had no significant effect on BW among the groups, as shown in Table 2. The possible reason is that the BW of growing geese did not increase significantly after 13 wk of age, although the weight of feathers increased weekly; hence, there was no significant correlation between their feather weight and BW.

Heavier live weight, longer necks and trunks, and deeper chests in male native Turkish geese might cause these differences in feather and down production (Saatc et al., 2007). Tilki et al. (2005) showed that the difference in live weight between the sexes became statistically significant at 6, 8, 10, 12, 14, and 16 wk of age. In this study, the BW, back length, sternum length, and chest girth of males were significantly higher than that of females during 13 to 25 wk of age (Table 2). The paw weight, heart weight, and the percentage of the paw and heart weight of FDBW of males were significantly higher than that of female geese (Table 3). The weight and body size of male geese were than those of female geese.

According to several reports, male geese have more feathers and down than females (Tóth et al., 1988; Kozák et al., 1997). Yildiz et al. (2009) suggested that female geese have intensive vexilla because of their barbs which are thinner than those of male geese. Kozák (1997) indicated that the correlation between the quantity of raw feathers and the percentage of down is significant but with a negative regression value in the case of males. In one previous report, the weight of 100 down feathers sampled from the third feather harvesting was 0.136 g for the layer and 0.143 g for the ganders on an average (Kozák et al., 1999). In this study, the dry feather weight of males was heavier than those of females (289 vs. 257 g). In the males, >10 cm feather weight and 4 to 10 cm feather weight were significantly higher than that of females (Table 4). On the other hand, <4 cm feather weight percentage of dry feather weight of male geese was significantly lower than that of females. It could be possibly because male geese have larger body size and surface area, hence, the heavier weight of feathers than that of female geese.

A comparison of down feathers from 14 to 16 and 22- to 24-wk-old geese has revealed that the weight of 300 down increased from 0.308 g to 0.349 g, making the difference between the 2 groups corresponds to 13%. Male geese had a heavier feather weight than females during 12 to 16 wk of age (Tilki et al., 2005). Saatci (2008) showed that feather weight was heavier in 10-wk young geese, and the older the goose a higher content of down, showing that down hadn't reached maturity in young geese. Van De Wetering and Cooke (2000) indicated that the mean daily change in primary feather length was 2.6%, which is consistent with rates reported for other waterfowl species. Schneider (1995) reported that after slaughter, 90 to 220 g of marketable feathers can be obtained per goose of 9- to 30-wk of age. In this study, the weight, percentage of dry feather weight, and FDBW of 4 to 10 cm feather had increased with age, and this may be the primary reason for the increase in the weight of feathers. The proportion of down also increased with the age of the geese (56–75%). Goose down is the most valuable feather and has a warm effect; the above-mentioned information shows that the older the goose had a higher content of down.

Tóth et al. (1988) indicated that the BW could be estimated between the quantity of feathers and the down. BW affects the total amount of feathers because plumage constitutes about 6.2% of BW (Szado et al., 1995). Saatci (2008) showed that the native Turkish geese have a total of 5.96% of the feathers of the BW; the correlation coefficient between live weight and down weight was 0.55. Similarly, Tóth et al. (1988) indicated a correlation coefficient of 0.50, and Szado et al. (1995) reported that of 0.56. Saatci (2008) reported the importance of chest measurements in both correlations between the feather-down weights and body measurements. The strongest correlation was found between total feather yield and chest girth (0.75). Kozák (1997) also indicated a significant correlation between live weight and raw feather weight in gander (*r* = 0.40–0.62) and goose (*r* = 0.30–0.50). They showed that the total amounts of feathers of BW were about 4.28 to 5.82% in 13- to 25-wk-old White Roman geese. The correlation coefficients between live weight and feather characteristics were not significant (Table 5); this may be because the White Roman goose did not differ in weight after 13 wk of age, so it is not related to the feather trait. The 18-h FDBW and down percentage had a significantly negative correlation in grower geese (−0.55, Table 5). Likewise, the 18-h FDBW and 4 to 10 cm feather weight had a significantly negative correlation in grower geese (−0.42). The reason for higher FDBW with the 4 to 10 cm feather weight may be due to no change in BW but the continuous growth of feathers.

## CONCLUSIONS

This study revealed that the age had no influence on BW among the 13- to 25-wk-old geese, and a good down percentage and dry weight were observed in 25-wk-old geese. The male geese had a lower (<4 cm) feather weight percentage of dry feather weight than female geese. The 18-h FDBW and down percentage were significantly negatively correlated in grower geese.
